# A Critical Review of EU Key Indicators for the Transition to the Circular Economy

**DOI:** 10.3390/ijerph18168840

**Published:** 2021-08-22

**Authors:** Roxana Lavinia Pacurariu, Sorin Daniel Vatca, Elena Simina Lakatos, Laura Bacali, Mircea Vlad

**Affiliations:** 1 Institute for Research in Circular Economy and Environment “Ernest Lupan”, 400609 Cluj-Napoca, Romania; roxana.pacurariu@ircem.ro (R.L.P.); laura.bacali@mis.utcluj.ro (L.B.); mircea.vlad@ircem.ro (M.V.); 2 Department of Management and Economical Engineering, Faculty of Machine Building, Technical University of Cluj-Napoca, 400114 Cluj-Napoca, Romania; 3 Plant Physiology Department, Faculty of Agriculture, University of Agricultural Sciences and Veterinary Medicine, 400372 Cluj-Napoca, Romania

**Keywords:** circular economy, sustainability, supply chains, consumption

## Abstract

The objective of this paper is to analyze the extent to which the system of indicators that is used in the Monitoring Framework for the transition to the circular economy (CE) is efficient and relevant in their contribution to the sustainable development of European communities. The fundaments of the transition framework and the main characteristics of the circularity indicators are presented. A critical review was performed in order to fulfill the objective of analyzing the current indicators. It is concluded that the indicators in the current framework are (as a selection from a very broad range of indicators theoretically proposed and with estimated practical applicability) limited from the perspective of circularity only to waste generation and recycling processes containing recyclable materials, without including important circularity indicators related to the prolonging and extending the life cycle of products and materials. This paper proposes and defines such an indicator, based on the consideration of the fundamental scalars describing economy, mass, energy, time, and value, respectively. The indicator is described and its applicability in all the phases of the economy is estimated.

## 1. Introduction

The concept of circular economy (CE) is currently a “fashionable” concept, still far from being complete, cursive, and completely defined [1]. Being one of the main building blocks of sustainability, mainly related to development, circularity is a much newer feature of a new economic paradigm [2]. Circular economy features a focused interest in minimizing the economy inputs and losses, in enhancing and preserving the natural capital, and also in increasing the efficiency of economic processes in the management of finite resources [3]. In fact, the use of the terms “linear economy” and “circular economy” does not appear to be the most appropriate. The circular economy can be considered an alternative that can co-exist with the linear economic system, as the transition to the circular economy can be made by expanding, along a spiral of processes, the “linear” economy.

Therefore, the circular economy is an umbrella type concept [4,5,6] that when put into practice has the effect of minimizing the environmental and societal impacts of human activities and stimulates sustainable growth, based on the conservation of stocks of resources (including slowing raw material inflows and minimizing waste generation) [7,8,9]. The circular economy can be operational at a microeconomic level (products, economic operators, consumers), mesoeconomic level (industrial parks), and macroeconomic level (locality, region, country etc.), aiming at generating sustainable development, simultaneously with economic prosperity and social equity and inclusion, for the benefit of present and future generations [1].

We consider that the above conceptualization is fully applicable for the period of transformation of the economy through circularization. The transition process will end with a new concept of economy, which will incorporate separate and new concepts such as “green” economy and bioeconomy [10].

Based on the vision of the alternative offered by the circular economy aimed at sustainable growth, the European Union (EU) made huge efforts to establish, since the initiation of the transition, a monitoring framework for progress. The actual framework for monitoring selected relevant and applicable drivers and indicators for transition. The selection took into consideration not less than 364 indicators for the circular transition [2,11]. Indexes were proposed, together with new methodologies for calculation [12,13], and assessment methodologies and tools that were already in use [2,14,15,16,17,18,19]. 

Because of the complexity of the circular economy concept and the vast influence it exerts on so many areas of major importance, more and more stakeholders come to realize that a monitoring framework for the transition to the circular economy with a high degree of adaptability is a real need for European communities. Once it was defined at the level of the European Union, the circular economy concept was accompanied by the intensification of the political, regulatory, technical, and scientific activity at the level of the entire union.

Specifically, in 2015 [20], the European Commission (EC) adopted an ambitious ‘Circular Economy Package’. The EU Action Plan established a program of actions and measures that covers the entire life cycle of the product, from production and consumption, through waste management to the secondary raw materials market. As part of the Action Plan and its ongoing efforts to transform the EU economy into a sustainable economy and for the implementation of this plan, the EC adopted, in 2018 [21], a new set of measures.

The new measures also included a Transition Monitoring Framework [22], applicable at the level of each Member State and at the level of the entire EU. By establishing the Monitoring Framework, the Action Plan is enriched with the necessary means to monitor progress. Therefore, it was necessary to develop a system of indicators that allows the evaluation of progress. Although the indicators included in the system mostly use data that is already collected, efforts are made on taking the necessary steps to ensuring the improvement of the quality of the collected data. The generated system of indicators completes the dashboard on resource efficiency and the dashboard of raw material resources, which have been implemented in recent years by the European Commission.

Therefore, the transition to CE requires changes at micro (resources, products), meso (sector, supply and value chains), and macro (economy, ecosystem) scales. One of the major gaps in the current research is how to identify and assess effects of the CE initiatives and indicators from a systemic perspective [23,24,25]. To address this gap, extensive research is needed to explore the effects of CE in an integrated way, but also to provide robust evidence of concrete opportunities and challenges for better design and methodologies. Our main research question addresses to what extent the system of indicators used in the Monitoring Framework for the transition to the circular economy is efficient and relevant in its contribution to the sustainable development of European communities. Therefore, our main objectives were as it follows: (i) to explore the extent to which the current Monitoring Framework is feasible when considering the whole definition of circular economy; (ii) to analyze if and how the time factor is considered in the context of circular economy indicators; and (iii) to propose a new global indicator that can facilitate the way towards circularity.

## 2. The Methodology and Concept of the Study

**Figure 1:**
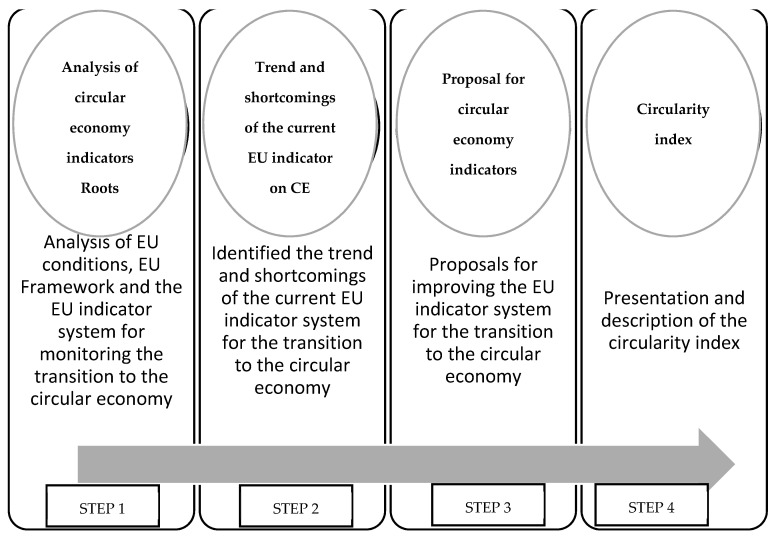
The research designing for analyzing circular economy (CE) indicators. Step 1: [7,8,10,12,13,14,15,16,17,18,19,20,21,22,23,24,25,26,27,28,29,30,31,32,33,34,35,36,37,38,39,40,41,42,43,44,45,46,47,48,49,50,51,52,53,54,55,56,57,58,59,60,61,62,63,64], Step 2: [2,5,51,52,53,54,55,56,57,58,59,60,61,62,63,64,65,66], Step 3: [37,67,68,69].

The present research was based on a four-step process aimed at developing the calculation of a circularity index, as seen in Figure 1. The concept presented below is original and was developed by the authors for achieving the stated goal of the research based on “A new, consonant approach of circular economy based on the conservation of the fundamental scalars of physics” [26], which was developed by the authors (for a previous study). The concept relies on the idea that in order to describe any kind of economic development, in a unitary, circular approach, it is essential to differentiate between the degenerative (“linear”) side of the economy and the regenerative and restorative side. The degenerative portion has its own adjustments, developed over time, to ensure the efficiency and profitability of direct activities based on specific indicators (turnover, specific consumption, productivity, profit, life, built-in energy, inflation, environmental impact, human resources, capital etc.), and of some indices validated in practice and which will maintain their use. These settings are defined and applied based on hereditary economic genomes. Both physical and value (monetary) units of measurement are used. The degenerative (linear) portion is characterized by its preference to break open the closed natural cycles (including optimal use of inputs, energy and material recovery inside value-adding processes, loss reduction etc.). In fact, we can associate the process of “circularizing” the economy with material regeneration (abiotic and biotic), renewable energy, controlled intervention in the natural cycles, extension of the product life, and reduced impact on climate metabolism. The external adjustment loop is supported by the actually named integrated waste management [4], but more adequately named Preparing of Waste for Reuse and Recycling. One can thus speak of an active side (constructive of new materials and products/services, a true synthesis of new molecules in economy) of the economy, and of a reactive portion (life-expanding, regenerative, de-constructive), which encompasses all the included regulation loops.

Therefore, in the following paragraph the foundations that formed the basis of the conceptual development of the index are presented:

Step 1. First, analysis of the circular economy indicators used globally was carried out, focusing on the degree of compatibility for use in EU conditions. The EU Framework for monitoring the transition to circular economy and the EU indicator system for monitoring the transition to the circular economy were examined as well. A bibliographic search was carried out in the Web of Science database, using the terms “Indicators” and “Circular Economy”. This initial search identified 870 articles that contained those terms in the title, summary, or keywords.
Step 2. In this step, we identified the main features of the current trends and limits at the micro-level (resources, products,), meso-level (sector, supply and value chains), and macro-level (economy, ecosystem) concerning the development of EU indicators targeted at facilitating the transition to the circular economy. The following inclusion criteria were used: (i) articles published between 2010–2020; (ii) articles that present an application/description/analysis of indicators in the field of Circular Economy and (iii) articles that discuss, analyze, or propose new indicators for measuring circular economy. Non-indexed studies, conference articles and book chapters were excluded. In addition, studies without full text or duplicate articles were excluded as well.
Step 3. This step involved analyzing the research done in the first two steps and developing proposals for improving the EU indicator system for the transition to the circular economy.
Step 4. The final step involved the conceptualization and presentation of the circularity index.

## 3. The EU Framework and Indicators for Monitoring the Transition to Circular Economy

The EU monitoring framework for the circular economy, released in 2018 [21,22] includes ten key indicators, covering every phase of the product life cycle, but also the major aspects of competitiveness. All indicators are updated regularly, their values being available on a dedicated internet domain. The taxonomy and the description of the indicators are presented in the document presented by the commission, as an annex to the Communication from the Commission to the European Parliament on the monitoring framework for the circular economy. 

The establishment of the monitoring framework and indicators has, for the current phase of the transition, the following basic directions:

(a) Direct, intrinsic monitoring of the transition through the soil resources used in the production and consumption of goods (products and services), having as objective their conservation;
(b) Indirect (extrinsic) monitoring of the consumption of other economic resources associated with production and consumption, with the aim of conserving them;
(c) Monitoring the achievements obtained as a result of the politically established strategic objectives, based on the registered results and their dynamics;
(d) Monitoring the impact (effects) of the launched actions, regarding sustainable development, as well as propagated effects of the material conservation on the natural and the socio-institutional environment.

The transition monitoring process and the indicators currently used characterize a first stage, of incipient transition, and the elaborated strategies focus on monitoring the material resources. Through this limitation, the considered effects are either direct (conservation of materials) or propagated (impact on the natural and socio-institutional environment). This limitation will have to be overcome once the initiation phase is completed and the preparation of accelerating the transition, within a growth program, is long-term. This program must have in each phase of its conception and application, a rough declarative inventory of what is to be measured and what can be measured.

The system of indicators must identify what is being measured, how the measurement is performed (method, data sources, and units of measurement), what are the relevant information extracted, and they must establish and update when (at what intervals) the measurements are performed, so that the process is controllable and the application of decisions is not made late or too fast. In addition, it is necessary to identify who is responsible for measuring and applying the respective decisions.

From the beginning, it is important to mention that establishing the basic principles that the associated indicators must follow during the transition to the circular economy is an important step in defining the growth model of the circular economy. From the study of the academic literature, we can deduce a set of principles that govern, at this moment, the substantiation and application of an appropriate transition monitoring framework, accompanied by robust and easy-to-use indicators. The current indicators are presented in Table 1, together with the field of application, type, conservation (the manner in which the indicator prevents the wasteful use of a resource) and the strategies they address, in accordance with the taxonomy developed by Bocken, De Pauw, Bakker, and Van Der Grinten [27]. 

### 3.1. The Circularity of the Economy Is an Integral Part of Sustainable Development

Recent literature [28,29,30,31,32,33] pays great attention to the relationship between sustainability and circularity. The analysis takes into account the effects that a circular economy has on sustainable development. Often, the effects are addressed to the three essential components of sustainable development, specifically the environmental dimension, but also the economy of resources and the balance of society (socially and institutionally). 

From this perspective, it becomes clear that monitoring the transition to a circular economy has to be based on indicators that will be used for analysis and monitoring of sustainability. The current monitoring framework, proposed at an EU level, contains eight indicators (Table 1) (out of ten) that are among the sustainability monitoring indicators.

Therefore, ideally, the monitoring of circularity and its system of indicators must be found in the system of indicators for sustainability, overlapping being a condition. Undoubtedly, in the specific context of the monitoring framework for the transition to the circular economy, specific indicators will be generated and used, corresponding to the detailed action plans for this process.

### 3.2. Reducing the Consumption of Raw Materials Is Essential

Monitoring currently limits the measurement of circularity in relation to the material resources of the soil, separate from the other resources gathered in the production and consumption processes, at least for the current period of evolution of the concept and comprehension of the circular economy. As a result, any action will have to be measured directly by indicators related to the flow of materials (and associated energy)—for inputs, for actions, for outputs, for results, and for effects. Analysis of the dynamics of achievements is important.

In fact, indicators for reducing materials are currently used as a indicators of sustainability as well. The monitoring framework created for the EU can be viewed as an extension of the UN indicators [34,35,36]. Reducing the consumption of raw materials in the soil should also include the raw materials used to generate the energy needed to transform raw materials through all activities associated with the economy. The main character of these indicators in the launching stage and ifn the first phase of accelerating the transition is generated by the level of achievements that are already adopted regarding the consumption of raw materials and materials for the years 2030 and 2050 [37].

### 3.3. The Inclusion of All Resources Is Essential for Their Conservation

Throughout its history and evolution, the economy has distorted and continues to distort the natural cycles of equilibrium in nature. The balance between these cycles can be achieved, first of all, by reducing/eliminating dangerous and toxic substances and by generating new, intelligent chains, including the replacement of abiotic materials with biological ones.

The use of this principle [38,39,40] allows the determination of performance indicators and dynamics of the transition process to the circular economy, based on energy, and water–air management. 

During the transition period, the use of direct and coordination indicators is a condition for verifying the sustainability of specific policies, strategies, and actions. The most important natural cycles that benefit from coherent and continuous reporting data are: the water cycle, the air cycle, the energy cycle, and the human resources cycle. These cycles in turn have an impact on natural biological cycles [41,42,43].

As the economy has as an essential part in the production of goods, and goods include not only material resources, but also energy, water, airborne substances, human, and biological resources, circularity must express the processes of storage, preservation (storage), and related regeneration/disposal. Therefore, a circularity analysis must also be based on indicators of energy, water, air, human resources, and biology. All the more so as these resources are either incorporated in products/services, or auxiliary consumed, without being incorporated [43,44,45,46].

### 3.4. The Compatibility of the Monitoring Process with the Policy Evaluation Schemes

**Figure 2:**
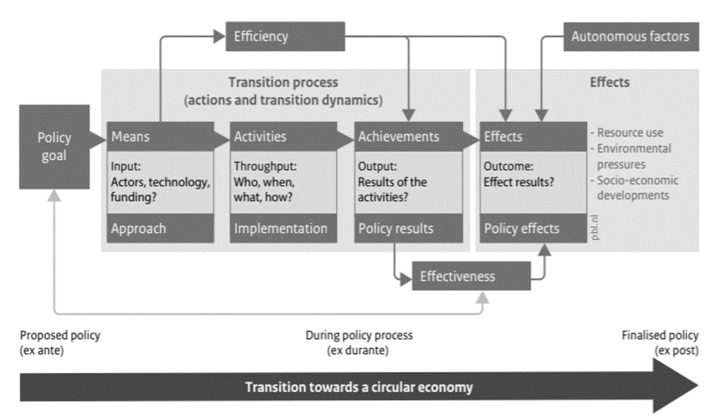
Policy assessment framework for measuring the progress of the transition towards a circular economy by Potting, Hekkert, Worrell, and Hanemaaijer [30].

A good monitoring process must be compatible with the strategies and political action plans and to ensure the coordination of the components of the transition over time, respectively, as in Figure 2: (i) Actions included in political programs; (ii) Transition dynamics; (iii) The effects on the consumption of natural resources, the natural environment and the national-economic environment. It is also expected that an overlap of the indicators is used for the general policy of the governments and the specific ones, on priority sectors and, within them, on product/material groups [47].

At a general level, the implementation of transition strategies (having the proposed results and achievements) results in desired, programmed effects on development (use of resources, environmental protection and socio-institutional balance). As a principle, the transition process results from the implementation of the chosen strategies (main achievements), which will result in obtaining the desired effects [48].

The monitoring must be performed in correspondence with the general scheme presented below. 

### 3.5. Final Remarks on the Stage of Initiating the Transition to the Circular Economy

The analysis of the current stage, of initiating the transition, allows the elaboration of the following main conclusions:

(a) Policies, strategies, and action plans are in the process of being developed, evaluated, and updated. This involves changes and adaptations at relatively short intervals;
(b) At the EU level there are priority policies, strategies, and action plans that should be coordinated with the Circular Economy (within the current conceptual limits). The package of documents on the circular economy (including the Action Plan and the Monitoring Framework and the Green Action Plan for SMEs) correlates directly with the Roadmap on resource efficiency, the 7th Environmental Action Plan, with the Roadmap on the efficiency of resources, with the initiative on raw materials and with the regulations on green procurement;
(c) The process of developing robust, easy-to-use and consistent indicators used for monitoring is ongoing. The development of the indicator system is done by establishing indicators at macro level, which can be disaggregated at meso and micro economic levels, by sectors, organizations, but also by materials etc., as well as by aggregating some indicators at micro level, including by taking over some composite indicators [14,49,50,51,52] starting from monitoring at micro and meso level, for their use as macro indicators;
(d) The system of indicators should be developed harmoniously, in an multilevel approach. By disaggregation by priority sectors and, within them, by groups of products/services, overlaps can occur and contradictory values can be generated in the periodical evaluation process. The initial indicators, established on the occasion of the evaluation that establishes the core of departure data (and the associated values of the indicators) must be verified from the point of view of their generic character (both basic and track indicators -subindicators), the level is established to which they are applicable. The process of improving microeconomic indicators must be closely related to the development and use at this level of indicators and indices based on the decomposition (theoretical, but also practical, industrialized) of products, components and materials into components, materials, energy etc., based on the life cycle analysis, unitary applied and extended compared to the level of the current standard. These indicators and indices must then be checked for integration, by aggregation at the higher level in the system of macroeconomic indicators. Disaggregation of macroeconomic indicators and aggregation of microeconomic indicators, must be covered simultaneously, the result being a coherent system, as simple and effective as possible. The use of new methods, based on nodal and network analyses [32,49], which allow harmonious aggregation/disaggregation is an immediate task of research and policies generated/updated;
(e) The propagated effect (from the political objectives to the desired effects) must be studied carefully, in a nodal (network) approach as it constitutes a solid basis for evaluating not only the necessary resources, results and achievements and effects but especially the dynamics of the process of circularization of the economy. It must be considered that the direct effect of an objective (for example, reducing the consumption of raw materials) will have an obvious direct effect, measurable by the quantities of raw materials reduced, but also a propagated one, in other areas of development for example in gas generation, greenhouse effect (depending on the technologies throughout the life cycle), in employment, in the reorganization of institutions etc.
(f) The current indicators are specific for material flow, but are still incomplete. The evolution of input of non-renewable raw materials used to generate the necessary energy to transform the raw materials into products is not taken into consideration, nor the replacement of raw materials;
(g) There is no consideration of the time factor, which could substantially modify the economy when considered;
(h) The indicators should be applicable also to services, as for example, health services, where the stock and use of materials/substances is important and specific, as well as the energy use [53,54,55,56].

## 4. Results: Trends and Shortcomings of the Current EU Indicator on the Circular Economy

The current system of proposed indicators is based on a still unfinished definition of the circular economy and a narrow vision of the transition process, essentially focused on conserving natural material resources (reducing its consumption by reducing consumption, using secondary materials), which has the effect of reducing material waste and of environmental contamination, but also the creation of jobs on the regenerative part of the economy and the accentuation of the role of innovation [57,58,59].

The new trends are related, on the one hand, to political factors, which seek to hold producers responsible for waste, and thus, companies collaborate in the reuse of materials and packaging, on the other hand, consumer brands are increasingly exploring refilling models and the recycling of chemicals at a commercial scale, with increased attention to toxic chemicals in discussions about the circular economy. So we should see more such actions in the coming years.

Under these conditions, there was a tendency to reduce the system of possible indicators, proposed internationally, to a smaller number of key indicators [60]. Designed for macro-evaluations (national, European, or regional) and for the necessary comparison between regions and/or countries, the current system of key indicators (generic) allows the fulfillment of the role of awareness, measurement, and influence of political decisions in a rather vague manner, resembling the system of indicators for sustainability.

The system of the ten key indicators in the European Union (some being decomposed into secondary indicators) is designed for a periodic update, in line with the perceived progress of the circular economy and the need to refine the evaluations. The current system of indicators represents the basis of the analysis carried out at European Union level regarding the implementation of the Action Plan for the Circular Economy, but also the elaboration of current reference reports.

In its current proposal, the monitoring framework establishes (at the macro level) a basis for comparing progress, involving what can be measured at this time versus what would need to be measured. The issue of completing the framework remains open, as more relevant indicators can be evaluated and integrated. The following conclusions can be drawn regarding the shortcomings of the current monitoring framework:

(a) The current framework is based on a strict definition of the circular economy and does not address the monitoring of strategies and actions to extend the life of products. Closing the cycle, however, involves not only their recycling, but also actions designed to extend life—maintenance, repair, remanufacturing, based on a design for circularity), but also important steps to regenerate resources (abiotic or biotic). Regeneration corresponds at least to the quantitative recovery and composition of a natural resource. Instead, the monitoring framework should be based on the broad definition of economics [36] as an economic model in which planning, procurement, production, and reprocessing are designed and managed, both as a process and as a result, to maximize systemic functioning and human well-being. Therefore, we consider that in the monitoring framework, indicators should be introduced regarding resources, environment, and society, both, which should be methodologically related to economic processes, and will be translated into sustainability analyses.
(b) The current framework mainly refers to the saving of solid material resources and does not take into account the other resources gathered in the design, production, consumption, and final treatment of the product (land, water, air, energy, human resources, bio resources). Also, the references are related to the raw materials and the materials incorporated in the product, without extension to the product itself [62,63,64,65,66].
(c) The current framework does not take into account the conservation of product functions. This aspect is little studied, although there are a few studies analyzing indicators addressing the conservation of product function [67,68]. The problem is being studied everywhere, as it involves a new consideration of the law of supply and demand (overlapping supply and demand cycles and harmonizing their rotation) in terms of consumer behavior change, determined by resource limits, environmental pollution, and reconsideration of the professions and the value of the labor force.
(d) The EC monitoring framework contains mostly indicators that refer to the conservation of raw materials and materials, based on recycling and waste production. Material resources and waste are considered to be the exclusive focus of European transition policies [69]. This, in the conditions in which in the EU grouped strategies are elaborated, is in order to preserve the functions, products, components, and other resources incorporated or consumed by the audience. From our literature analysis, we observed that authors who develop indicators of the circular economy on a microeconomic scale are less concerned with conserving the energy embedded in products and evaluating waste generation. Energy recovery is often seen as the last applicable option. The Material Circularity Index [70] considers energy recovery as well as the amounts of non-recoverable waste and, therefore, may have an increased relevance. However, the influence that waste quality has on value conservation is rarely considered, although waste quality has a strong impact on recycling.
(e) The current framework contains both indicators for which reference data are established (both by specific, quantitative targets and non-specific, qualitative targets, but also indicators for which targets are not (yet) set. The consulted literature allows to highlight reasonable criticisms on the current monitoring framework [71].

An important implication is that by overcoming these shortcomings, the circular economy potential for improving the quality of life in European communities can be enabled. First of all, one of the main purposes of the CE, as we already mentioned, is to reduce the use of primary resources while maintaining the highest value of materials and products through various processes (recycling, reuse of products, component, refurbish etc.) and to transition towards greater use of renewable energy sources. The pursuit of these goals can have many positive health implications, such that direct and indirect benefits arise from reducing the environmental impacts of manufacturing processes [72,73,74,75] and making cost savings in households and in the health sector [76,77]. For example, by applying the CE principles (designing out waste and pollution, keeping products and material in use), we can expect to see a reduction in the total amount of harmful substances in the waste stream in the long term [78]. If these actions are successful and their broader consequences such as impacts on livelihoods are considered, they will cut health impacts and could benefit the poor, since the local and worker populations of unregulated dump sites would disproportionately experience these benefits.

## 5. Proposals for Improving the EU Indicator System for the Transition to the Circular Economy

The circular economy and its associated system of indicators resulted from an extremely fast process of conceptualization, inventory, classification, and analysis. A complex and current inventory of specific EC indicators is currently being made, to which is added an exhaustive review and critique.

To conceptualize a relevant system of indicators, it is obvious to differentiate between the concept of circular economy and the one of circular economics. It means making the clear difference between economy and economics. Economy can be understood as the aggregate of all the arrangements for the transformation of matter, using energy, based on creation/innovation and knowledge in products and services (values) for exchange in temporarily defined markets. Economics is an organized body of knowledge that studies the behavior and activities of an individual, group, organization, nation etc. that are related to maximize the satisfaction of wants or advance the welfare, safe environment, and economic growth, by optimum production, distribution, consumption, and exchange of temporarily limited resources that have alternative uses/reuses [79,80,81].

From the various definitions of the circular economy, it is clear that what we name “circular economy” is indeed the vision of a regenerative economy, by intention, design, and application, able to be both efficient (in what concerns the use of resources, wealth, and grow), but also effective (regarding mainly the environment and society/individuals). Three complex actions are defined to transform the economy from linear (degenerative) to circular regenerative: (i) design out waste and pollution, (ii) keep materials and products in use, and (iii) regenerate natural systems [82,83]. 

Both of the aggregate of activities taken into consideration and of the associated indicators. The coexistence of the Cradle to Cradle, Circular Economy, and Life Cycle Analysis (named as “love triangle”) [84] is visible. They are completing each other, and cannot be used alone to monitor the progress, even if they are individually indispensable. 

The OECD Inventory of Circular Economy Indicators [85] collected 474 circular-economy-related indicators, between 2018 and 2020. Collected indicators belong to 29 circular economy studies, of which eight are applied at the national level, eight at the regional level, and eleven at the local level. It becomes very difficult and very specialized and subjective to select the most relevant indicators for a particular economy and to compare the economies between them afterwards.

As we stated in the chapter above, the EU framework is limited and pushes the idea that circular economy is mainly about resource efficiency. 

Life Cycle Assessment (LCA) is a science-based technique for assessing the impacts associated with entire product life cycles, standardized in the ISO 14040-serie, which despite its partial support for assessing circular strategies, has many historical limitations in the way that model raw materials and resource considerations (which often take the linear economy as the frame of reference) [86]. LCA community is actually far for being included with the decision-making factors. CA does not yet have consistent accounting for changes in stocks of resources respecting mass balance principles for modelling of open recycling glops, for all relevant resources and impacts, i.e., a full economy-wide LCA perspective [87,88,89]. Additionally, it lacks the transparency of assumptions, reliability of data, and critical interpretation of results and trade-offs between a globally agreed numbers of impact categories, e.g., through valuation, as suggested in ISO 14008. Time is not a factor that is taken into consideration, making the monitoring and evolution difficult, asking for collaborative interpretations and calculations. 

In these circumstances, in place of making an exhaustive study on the association, correlation and causal relationships between multiple specific indicators (depending on field of application and analyses and on economic action intended), the kind of studies for which there is plenty of literature in the last years [90,91,92,93,94], we propose a general indicator, based on considering the main effects that the activities driven by a circular economics are generating. This indicator has the attributes that are necessary to evaluate progress at each moment and at each scale (product, group of products, organization, company, region, country). This assumption is based on the fact that the main scalars featuring any action (mass, energy, time, and value) are linked into a general formula, which can be applied for existing data, protecting the confidentiality and rights of all the stakeholders involved [95,96]. The proposed indicator is simple, relevant, and opens a well-directed and controllable synergy for the “love triangle to work. 

The indicator is based on the definition of considering a general effectiveness of economy as **EF_CE_**.

**EF_CE_** = **M_v_**.**C_v_**/**Aec** [kg*currency/J*s] (1)

where:

**Mv** is the total virgin mass which enters into an economic process plus the mass of non-renewable materials used to generate the energy used to transform the mass [**kg**]; 

**Cv** is the commercial value of the output from the process [**currency**] and

**Aec** is defined as the economic overall ACTION, based on the activities related to the manufacturing of products. 

ACTION is defined as the product between the **Er** (renewable energy—from renewable sources) and **Tt** (one period in which the measured mass is transformed). The unit of measurement for Action is [J*s].

The relationship (1) above is applicable to each phase (which may be itself a closed or an open loop) of transformation (extraction, material manufacturing, component and product manufacturing, preparing for reuse and recycling, use/reuse and recycling, and regeneration). 

The time factor in the relationship may be used to harmonize these frequencies of substances extraction, conversion into desired materials, manufacturing, preparing for reuse and recycling, recycling/regeneration, since it follows the accumulation and dissipations of the stocks of matter (as maters/materials are conservable). **Id** ensures that the same virgin mass, conservable, links all the phases in a regenerative cycle. 

The indicators may be applied to data already existing in the statistics and/or reports and gives similar results with the complicated calculations of indexes, based on multiple and complex indicators. Moreover, facto time is introduced, allowing for a synchronization of the economical phases pf production-use-waste generation and regeneration.

As a result of the research made in time by IRCEM, the product between the total energy consumption for transformation of **Et** and **Tt** (indice **t** is for transformation) has a normal distribution for random, aleatory measurements, for a given process (of transforming mass and energy into products, in time, under a given organization and management), which is very useful in the estimation and evaluation of proposals and in monitoring of the achievements.

As far as the application for the inverse branches (feedbacks) is concerned, the same distribution should be applicable, since the processes are identical by structure and development. Even in the process of use, the mass of the objects remains the same (with small variations due to the replacement of used or obsolete components) as the product between the energy used and time (of utilization) shall show the same distribution. To demonstrate this, further research is still necessary, and they should be conducted for the processes associated with the conversion of mass into energy and vice-versa [97,98,99].

## 6. Discussion

The analysis, evaluations, and ideas presented in this article aimed to determine the level at which the system of indicators that are used in the Monitoring Framework for the Transition to the Circular Economy are relevant, robust, and precise. At the same time, the need for the current system of indicators to be completed in the current phase of initiation and preparation for accelerating the transition process was explored.

The EU Framework contains a limited set of indicators, despite the large number of indicators put into discussion, proposed, or analyzed. The main barrier in expanding the set of indicators towards other important processes of circular economy consists of the lack of sufficient relevant and measured data [100,101,102]. 

There is a quite low level of engagement of the companies, whatever their size, field of activity, and turnover in a circular assessment process, as the reports show [102,103,104,105,106]. Based on an extensive literature, having as object the elaboration and use of indicators for monitoring the transition process, the indicators included in the current monitoring framework were analyzed from the point of view of the representation of the transition policy and strategy, of the actions included in the action plan, transition, and the results of these actions. Corresponding to the circularization of the economy, the phrase “supply chain” must be replaced by the “supply cycle”, characterized by inputs, outputs, storage, and recirculation [107]. 

It was observed that the limitation of the current monitoring framework is only to the aspects related to the conservation of material resources (using indicators similar to those in the analyzes of the evolution of sustainable development), even if some indirect indicators try to complete the determination between a circular economy and sustainable development. This observation allows the conclusion that, at the current stage, only the narrow definition of the circular economy applies.

Even if it is found that the developed macro indicators can be disaggregated by priority sectors, and within them by groups of materials or at regional level, company, and even below, there are difficulties in correlating them with specific indicators that apply at the micro-economic and meso-economic level [108,109,110,111].

Specifically, the system of indicators used at the macro scale does not take into account the specific composition of the materials. It allows only a global assessment, in broad categories of raw materials and constituent materials of products. At the level of each product and organization, there must be an intimate breakdown, based on rigorous material lists specified by the manufacturers of the products and their components, into basic materials and raw materials [112,113]. The life cycle analysis and the index (composite) of material circularity must be available on the regenerative portion of the economy, for the intimate highlighting of the stocks in use, as well as for the decomposition/recycling activities. Collaboration between material processors, product manufacturers, and waste management organizations must be extensive, using new methods (blockchain, Big Data) [114,115,116,117]. 

Currently, the methodologies used to aggregate circularity indicators, broken down into all the main components of the product cycle, at the level of product, service, or company, are developed, but their approaches are dependent of the region/country and their calculation is incomplete, due to leaking of data [118,119,120]. It can also be concluded that the indicators in the current framework are limited, from the point of view of circularity, only to waste recycling processes containing recyclable materials and that important circularity indicators are not included in the European Commission’s strategy and action plan, related to prolonging as much as possible the life cycle of products and materials.

Another important remark concerns the circularity of the service industry. Since services are extremely important in the formation of GDP (Gross Domestic Product) and can also have a significant effect on the carbon footprint [121], and their circularity must be assessed and monitored with similar indicators. In the case of healthcare, the principles of the circular economy could easily make specific services more accessible. For example, the model “product as a service”, in which hospitals pay for the use of medical equipment rather than purchase it could stimulate manufacturers to optimize design for reuse [122,123,124,125]. Circular solutions could also be applied to medical waste, as most unused medicines are dumped in household waste bins and can contaminate the water supply by disposing of them in landfills. Bringing them into a closed circular loop could help reduce costs for public health systems and reduce the carbon footprint of production, and the logistical costs of reuse processes are substantially lower than the value of the medical products. However, this would depend on meeting safety concerns regarding contamination, deliberate handling, use of counterfeit medicines, or improper storage conditions.

## 7. Conclusions

In order to prepare for the acceleration phase, it becomes necessary to expand the systems of indicators, so that the measures and strategies for the circularization of the economy are pursued as a whole and not only unilaterally, through excessive simplification. In this sense, new indicators were discussed and considered necessary and feasible to be measured and integrated in the physico-chemical chains of the products [126,127]. Through this, it becomes possible to rethink the valorization of economic activities, including the money chain of the product/service cycle and the activity-money determinism, following the fine-tuning of the manifestation of natural laws (law of supply and demand) to support the process of circularization of the economy [128,129,130].

Based on the natural law of supply and demand, we conclude that, in parallel with the circularization of supply (beginning from producers), it is necessary to take into account the circularization of demand [131,132], as this last cycle imposed a rethinking and a redesign of the marketing, acquisitions, and even of the design methods, in parallel with the further stimulation of the innovation [133,134,135,136,137,138]. However, the circulation of the demand it is not detailed in this article, but further research should study this approach as soon as possible.

It is true that in the first phase of a transition process, there is a need of stronger control and that the frequency of measurement must be bigger. This control cannot be too complex in the absence of a very careful insight of the associations, correlations and causality between the activities to which indicators are assigned. The risks are substantial. 

Furthermore, it is necessary to differentiate between quantifying the progress by its main parameters (as we proposed here) and the progress towards the objectives set up through strategies, plans of actions, and measures. 

However, further research and studies are necessary to prove the relevance and precision of the proposed indicator for specific applications at different levels.

## Figures and Tables

**Table 1:** Monitoring indicators present in the current Monitoring Framework at EU level. Adapted from [21].

Indicator Name	Field of Application	Type	Conservation	Strategies Addressed
1. Independence from raw materials	Production and Consumption	indirect	material	Business model strategies for slowing loops: Access and performance model
2. Public green acquisitions	Production and consumption	direct	knowledge	Public Acquisition strategies, circular cities strategies
3. Waste generation	Production and consumption	direct	material	Design strategies to close loops: design for a technological cycle, design for a biological cycle, design for dis- and reassembly
4. Food Waste	Production and Consumption	direct	material	Design strategies to close loops: design for a technological cycle, design for a biological cycle
5. Recycling rate	Waste Management	direct	material	Design strategies to slow loops: design for recyclability, design for upgradability and adaptability, design for dis- and reassembly
6. Recycling/Recovery for specific waste streams directly	Waste Management	direct	material	Design strategies to slow loops: design for recyclability, design for upgradability and adaptability, design for dis- and reassembly
7. The contribution of recycled materials to meeting the demand for raw material	Secondary Raw Materials	indirect	-	-
8. Trade in recyclable primary materials	Secondary Raw Materials	indirect	-	Business model strategies for closing loops: Industrial Symbiosis Strategies, Circular Cities Strategies
9. Private investment, jobs and gross value added with reference to the circular economy sectors	Competitivity and Innovation	indirect	value	Circular cities strategies
10. Number of patents related to recycling and secondary raw materials as a representation for innovation	Competitivity and Innovation	indirect	knowledge	Design strategies to slow loops: design for recyclability, design for upgradability and adaptability
